# An OB-fold complex controls the repair pathways for DNA double-strand breaks

**DOI:** 10.1038/s41467-018-06407-7

**Published:** 2018-09-25

**Authors:** Shengxian Gao, Sumin Feng, Shaokai Ning, Jingyan Liu, Huayu Zhao, Yixi Xu, Jinfeng Shang, Kejiao Li, Qing Li, Rong Guo, Dongyi Xu

**Affiliations:** State Key Laboratory of Protein and Plant Gene Research, School of Life Sciences, Peking University, 100871 Beijing, China

## Abstract

53BP1 with its downstream proteins, RIF1, PTIP and REV7, antagonizes BRCA1-dependent homologous recombination (HR) and promotes non-homologous end joining (NHEJ) in an unclear manner. Here we show that REV7 forms a complex with two proteins, FAM35A and C20ORF196. We demonstrate that FAM35A preferentially binds single-strand DNA (ssDNA) in vitro, and is recruited to DSBs as a complex with C20ORF196 and REV7 downstream of RIF1 in vivo. Epistasis analysis shows that both proteins act in the same pathway as RIF1 in NHEJ. The defects in HR pathway to repair DSBs and the reduction in resection of broken DNA ends in BRCA1-mutant cells can be largely suppressed by inactivating FAM35A or C20ORF196, indicating that FAM35A and C20ORF196 prevent end resection in these cells. Together, our data identified a REV7–FAM35A–C20ORF196 complex that binds and protects broken DNA ends to promote the NHEJ pathway for DSB repair.

## Abstract (web summary)

How repair pathway selection occurs is still a matter of debate and many factors have been associated to this function. Here the authors provide insight into the role of FAM35A and C20ORF196, two REV7-interacting proteins, which are recruited at double-strand breaks to promote non-homologous end joining repair.

## Introduction

Mammalian cells utilize two major pathways to repair double-strand breaks (DSBs), which are the main source of DNA damage and must be repaired to allow cells to survive. One pathway depends on homologous recombination (HR), whereas the other one uses non-homologous end joining (NHEJ)^1,2^. Resection of DNA ends, which converts broken DNA into 5ʹ-recessed ends suitable for HR, is a key reaction by which the selection of the pathway is controlled^1,2^. BRCA1 promotes this reaction and thus stimulates the HR pathway^3^, whereas 53BP1 and its downstream proteins, RIF1^4–8^, PTIP^9^ and REV7^10,11^, suppress the reaction and thus facilitate the NHEJ pathway. However, how the 53BP1–RIF1–REV7 axis suppresses the resection is unclear.

Here, we identify a REV7 complex with FAM35A and C20ORF196, which is distinct from the Polζ complex of REV7. FAM35A resembles a telomere-protecting protein, POT1, in that both proteins contain three OB-fold domains and bind single-strand DNA (ssDNA). Cells lacking either FAM35A or C20ORF196 are largely defective in NHEJ pathway, but not HR pathway. Interestingly, the absence of FAM35A or C20ORF196 rescues HR and end resection in the BRCA1-deficient cells. Thus, our data identify a REV7–FAM35A–C20ORF196 complex that protects broken DNA ends to antagonize BRCA1-dependent resection, and its mode of action may resemble that of POT1.

## Results

### FAM35A and C20ORF196 form a complex with REV7

**Fig. 1:**
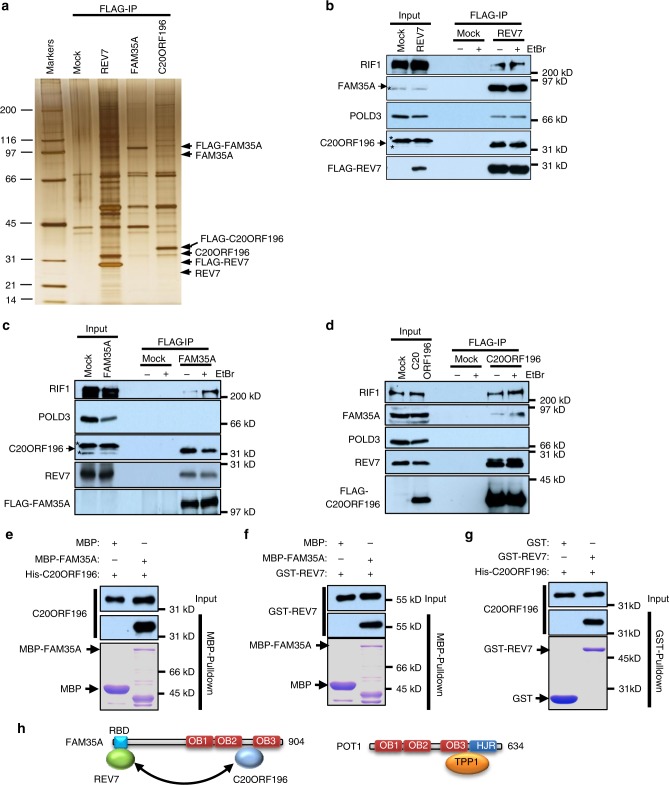
FAM35A and C20ORF196 form a complex with REV7. **a** Silver-stained SDS-PAGE gel showing the polypeptides that were immunopurified from extracts of HEK293 cells expressing FLAG-tagged REV7, FAM35A and C20ORF196 using the anti-FLAG antibody. The major polypeptides on the gel (arrows) were identified by mass spectrometry. **b**–**d** Immunoblot showing the IP of FLAG-tagged REV7 (**b**), FAM35A (**c**) and C20ORF196 (**d**). IPs were performed with or without 100 μg ml^–1^ EtBr. The cross-reactive bands were indicated as asterisks. **e**–**g** MBP-pulldown (**e**, **f**) and GST-pulldown (**g**) examined the direct interactions among REV7, FAM35A and C20ORF196. The bait and prey proteins were detected with Coomassie blue staining and immunoblotting, respectively. **h** Schematic representation of FAM35A and POT1

To investigate the mechanism of REV7 in protecting DSB ends, we immunopurified its complexes from HEK293 cells transiently expressing FLAG-REV7 with anti-FLAG antibody. Mass spectrometry analysis revealed that two proteins, FAM35A and C20ORF196, co-immunoprecipitated with REV7 (Fig. 1a, Table 1 and Supplementary Data 1). Immunoblotting confirmed this finding (Fig. 1b). Immunoblotting and mass spectrometry analysis of reciprocal immunoprecipitation using HEK293 cells expressing FLAG-tagged FAM35A and C20ORF196 showed that REV7, FAM35A and C20ORF196 are mutually present in their immunoprecipitates (Fig. 1a–d, Table 1 and Supplementary Data 1). REV7 is one component of the Polζ complex^12^. Mass spectrometry analysis of the immunoprecipitated mixtures and immunoblotting revealed that the Polζ subunits, REV3L, POLD2 and POLD3 existed in the complexes of REV7 but not those of FAM35A and C20ORF196 (Fig. 1a–d, Table 1 and Supplementary Data 1), suggesting that REV7 forms two distinct complexes: the Polζ complex and the REV7–FAM35A–C20ORF196 complex.

**Table 1:** Peptide number identified by mass spectrometry

Identified proteins	FLAG-IP			
	Mock	REV7	FAM35A	C20ORF196
REV7	0	25	5	3
FAM35A	0	3	88	12
C20ORF196	0	0	5	20
REV3L	0	1	0	0
POLD2	0	2	0	0
POLD3	0	3	0	0
RIF1	0	15	19	3

Full list of mass spectrometry data is available in Supplementary Data 1

To examine the direct interactions in the REV7–FAM35A–C20ORF196 complex, we performed MBP- and GST-pulldown assays with recombinant proteins. Results showed that MBP–FAM35A pulled-down both C20ORF196 and REV7 (Fig. 1e, f), and GST–REV7 also brought-down C20ORF196 (Fig. 1g), suggesting that these three proteins are able to interact directly with each other.

In addition to REV7, FAM35A and C20ORF196, mass spectrometry also identified RIF1 in all the immunoprecipitates of these three proteins (Table 1 and Supplementary Data 1). Because RIF1 is a common contamination in immunoprecipitations^13^, we examined this finding carefully under a condition with ethidium bromide (EtBr), which impairs protein–DNA interaction. Immunoblotting confirmed that RIF1 was co-purified by FLAG-tagged REV7, FAM35A or C20ORF196 even with the presence of EtBr (Fig. 1b–d). These results suggest that RIF1 interacts with the REV7–FAM35A–C20ORF196 complex not through a bridge of DNA.

### FAM35A prefers to bind ssDNA

**Fig. 2:**
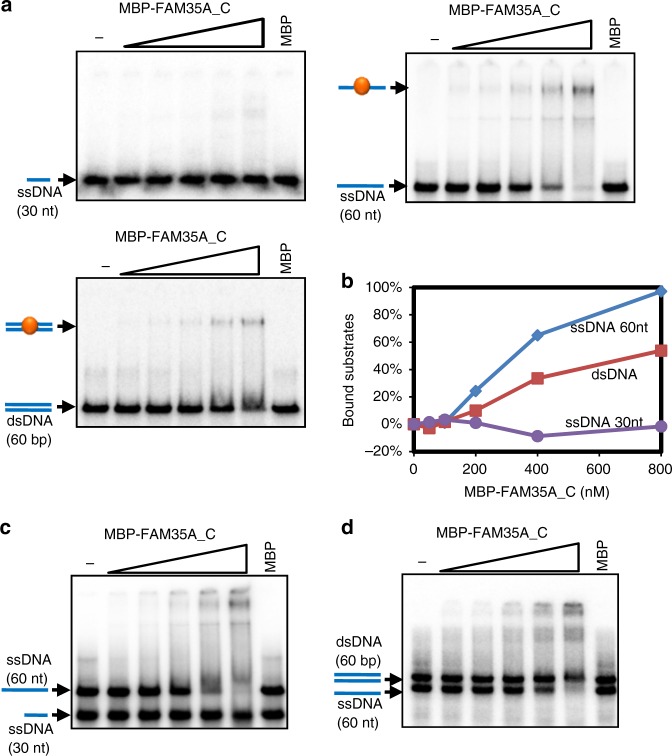
FAM35A prefers to bind ssDNA. **a**, **b** Gel-shift assay (**a**) and its quantification (**b**) shows that MBP-FAM35A_C (381–904 aa) prefers to bind ssDNA. Reactions contained 5 nM of the indicated ^32^P-labeled substrates and 0, 50, 100, 200, 400 or 800 nM purified MBP-FAM35A_C or 800 nM MBP. **c**, **d** Competition experiments between 60 nt ssDNA and 30 nt ssDNA (**c**) or 60 bp dsDNA (**d**) for MBP-FAM35A_C binding

FAM35A and C20ORF196 are expressed in most vertebrates and a few of invertebrates. Three-dimensional structure prediction with HHprep (https://toolkit.tuebingen.mpg.de/#/tools/hhpred) revealed that while C20ORF196 has no recognizable domains, FAM35A contains three OB-folds at its C-terminus, which resemble the structure of POT1, a component of the telomere protection complex (Fig. 1h and Supplementary Fig. 1a, b). It has been shown that many OB-folds are ssDNA-binding domains and/or protein-interacting domains^14^. In fact, POT1 has been shown to bind ssDNA regions of telomeric DNA through its OB-folds and this binding is required for telomere stability^15^. The similarity between FAM35A and POT1 prompted us to examine whether FAM35A may bind ssDNA and protect broken DNA ends. We expressed and purified a recombinant protein containing all three OB-fold domains (381–904 aa) fused to the maltose-binding protein (MBP-FAM35A_C; Supplementary Fig. 2a, b). Gel-shift assay showed that MBP-FAM35A_C bound a long ssDNA substrate (60 nt) but not a short one (30 nt; Fig. 2a, b). It also bound double-strand DNA (dsDNA; 60 bp) but with a much lower affinity than ssDNA (60 nt; Fig. 2a, b), with a *K*_d_ of ~ 750 nM, compared with ~320 nM for 60 nt ssDNA. We further carried out competition experiments and confirmed that MBP-FAM35A_C prefers to bind 60 nt ssDNA compared with 30 nt ssDNA and 60 bp dsDNA (Fig. 2c, d).

### FAM35A interacts with REV7 through its N-terminal region

FLAG immunoprecipitation and MBP-pulldown showed that a conserved N-terminal region RBD (REV7-binding domain; 1–54 aa) is necessary and sufficient for its interaction with REV7 (Supplementary Fig. 3a–e). Mutation in two conserved prolines to alanines (AA, P14A/P17A) of RBD region completely disrupted its interaction with REV7 (Supplementary Fig. 3a, f). In contrast, deletion of the N-terminal region of FAM35A (OB1-3) did not affect its interaction with C20ORF196 (Supplementary Fig. 3a, g). The further deletion of OB1 (OB2-3) strongly reduced its interaction with C20ORF196, suggesting that OB1 contributes to this association. Moreover, deletion of both OB1 and OB2 (OB3) or OB3 (OB1-2) completely disrupted its interaction with C20ORF196, indicating that the region (OB2-3) containing OB2 and OB3 are required for the interaction between FAM35A and C20ORF196 (Supplementary Fig. 3a, g, h).

### FAM35A and C20ORF196 were recruited to DNA damage sites

We observed that green fluorescent protein (GFP)-tagged FAM35A was recruited to laser-induced DNA damage sites, implicating that FAM35A may act directly at the site of DNA damage (Supplementary Figs. 3a, 4a). Although the GFP-FAM35A_RBD displayed strong signals at DNA damage sites, RBD domain-deleted mutant FAM35A_ΔRBD and FAM35A_OB1-3 only showed very weak signals (Supplementary Figs. 3a, 4a), suggesting that FAM35A may be recruited to DNA damage sites by two different mechanisms: the majority of FAM35A may be recruited by its interaction with REV7 through the RBD domain; whereas the minority of FAM35A may be through its OB-folds domain-mediated binding to the ssDNA at the broken DNA ends. These two mechanisms cooperatively recruit FAM35A to DSB sites because lacking any of them leaded to reduced signals (Supplementary Fig. 4b). Moreover, these OB-folds were able to be recruited to DNA damage sites individually (Supplementary Figs. 3a, 4c). Consistent with our speculation, depletion of REV7 strongly reduced the recruitment of GFP–FAM35A to DNA damage sites (Supplementary Fig. 4d, e). REV7 is recruited to DSB sites by RIF1^10,11^. Consistently, the recruitment of the RBD region, but not the OB1-3, of FAM35A was strongly decreased when RIF1 is absent (Supplementary Fig. 4f–h). We further observed that GFP–C20ORF196 was also recruited to laser-induced DNA damage sites (Supplementary Fig. 5a), consistent with that it forms a complex with REV7 and FAM35A. Interestingly, the recruitment of REV7, FAM35A and C20ORF196 to DSB sites are interdependent (Supplementary Fig. 5a–g), implicating that they may be recruited as a complex. Moreover, RIF1 depletion also strongly reduced the recruitment of C20ORF196 (Supplementary Fig. 5a). These results show that the REV7–FAM35A–C20ORF196 complex is recruited to DSB sites dependent on RIF1.

### FAM35A and C20ORF196 promote NHEJ to repair DSB

**Fig. 3:**
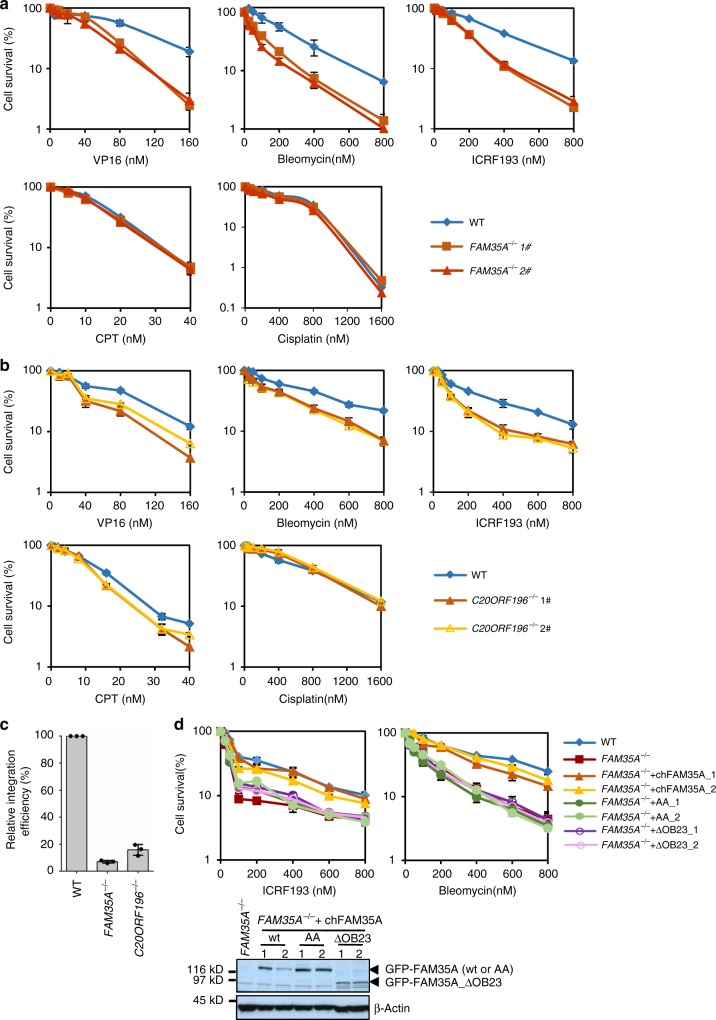
FAM35A and C20ORF196 promote NHEJ to repair DSB. **a**, **b** Sensitivity assay of FAM35A (**a**) and C20ORF196 (**b**) knockout DT40 cells. The mean and s.d. from three independent experiments are shown. **c** Random integration assay of FAM35A and C20ORF196 knockout DT40 cells. Cells were transfected with the pLoxPuro plasmid and clonogenic potential was determined in the presence of puromycin. Data are expressed as the mean number of puromycin resistant colonies ± s.d. (*n* = 3). **d** Sensitivity assay of *FAM35A*^*−/−*^ cells re-expressing GFP-fused wild-type or mutated chicken FAM35A. Protein expression levels of GFP-chFAM35A were shown by immunoblotting on the bottom

To investigate the function of FAM35A and C20ORF196 in vivo, we generated the *FAM35A* or *C20ORF196* null DT40 cells by gene targeting (Supplementary Fig. 6a–d). These cells displayed strong sensitivity to reagents that induce DSBs, including Bleomycin (a radiomimetic agent) and VP16 (etoposide, a Topoisomerase II [Topo2] inhibitor, which directly induces DSBs; Fig. 3a, b), demonstrating that FAM35A and C20ORF196 are required for normal DSB repair in vivo.

To examine which DSB repair pathway is defective in *FAM35A*^*−/−*^ and *C20ORF196*^*−/−*^ cells, we utilized two drugs that induce distinct DSB damages that depend on a specific pathway for repair: ICRF193, which is a Topo2 inhibitor and induces DSB damages that specifically require the NHEJ pathway^16–18^; and CPT, which is a TopoI inhibitor and induces one-end DSBs that depend on the HR pathway^16–18^. *FAM35A*^*−/−*^ cells were hypersensitive to ICRF193, but not CPT (Fig. 3a), suggesting that FAM35A functions in NHEJ but not HR pathway. Other than hypersensitivity to ICRF193, *C20ORF196*^*−/−*^ cells also showed mild, but reproducible, sensitivity to CPT (Fig. 3b), demonstrating that C20ORF196 has other function than NHEJ. NHEJ is essential for foreign DNA random integration in DT40 cells^4,19^. Consistently, random integration is decreased 14.2 and 6.3-folds in *FAM35A*^*−/−*^ and *C20ORF196*^*−/−*^ cells, respectively (Fig. 3c).

To examine which domain of FAM35A is required for its function in NHEJ, we carried out complementation experiments. Wild-type FAM35A, but not AA (P14A/P17A, which loses its interaction with REV7) and ΔOB23 (1–576 aa, which loses interaction with C20ORF196 and ssDNA), restored the resistance to DSB-inducing drugs, ICRF193 and Bleomycin (Fig. 3d), demonstrating that both the interactions of FAM35A with REV7 and C20ORF196 or ssDNA are important for its function in NHEJ.

**Fig. 4:**
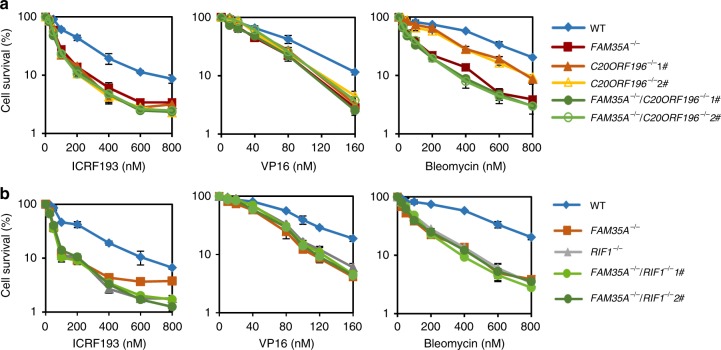
FAM35A and C20ORF196 are in the same pathway with RIF1 to repair DSB. **a**, **b** Genetic interaction analysis of FAM35A with C20ORF196 (**a**) and RIF1 (**b**) in DT40 cells. The mean and s.d. from three independent experiments are shown

To check if FAM35A and C20ORF196 work in the same DSB repair pathway, we generated *FAM35A*^*−/−*^*C20ORF196*^*−/−*^ double knockout cells and performed epistasis analysis. *FAM35A*^*−/−*^*C20ORF196*^*−/−*^ cells showed similar sensitivity to VP16, Bleomycin and ICRF193 as single knockout cells (Fig. 4a), demonstrating that FAM35A and C20ORF196 play a role in the same pathway to repair DSBs. REV7 suppresses end resection and promotes NHEJ downstream of RIF1^10,11^. We examined if FAM35A and C20ORF196 also act in NHEJ in the same pathway with RIF1. *FAM35A*^*−/−*^*RIF1*^*−/−*^ cells showed no more sensitivity to DSB-inducing reagents than single knockout cells (Fig. 4b), demonstrating that FAM35A acts in the same pathway as does RIF1. We thus concluded that REV7 forms a complex with FAM35A and C20ORF196 to repair DSBs through NHEJ.

REV7 is one subunit of Polζ, which is required for DNA inter-strand crosslink (ICL) repair^12^. REV7-deficient DT40 cells are hypersensitive to ICL-inducing reagents, such as cisplatin^20^. However, *FAM35A*^*−/−*^ and *C20ORF196*^*−/−*^ cells were not sensitive to cisplatin (Fig. 3a, b), suggesting that FAM35A and C20ORF196 are not required for the function of REV7 in ICL repair. These results are consistent with the biochemical data that REV7 forms a distinct complex with FAM35A and C20ORF196 from the Polζ complex.

### FAM35A and C20ORF196 block resection in the *BRCA1*^*−/−*^ cells

**Fig. 5:**
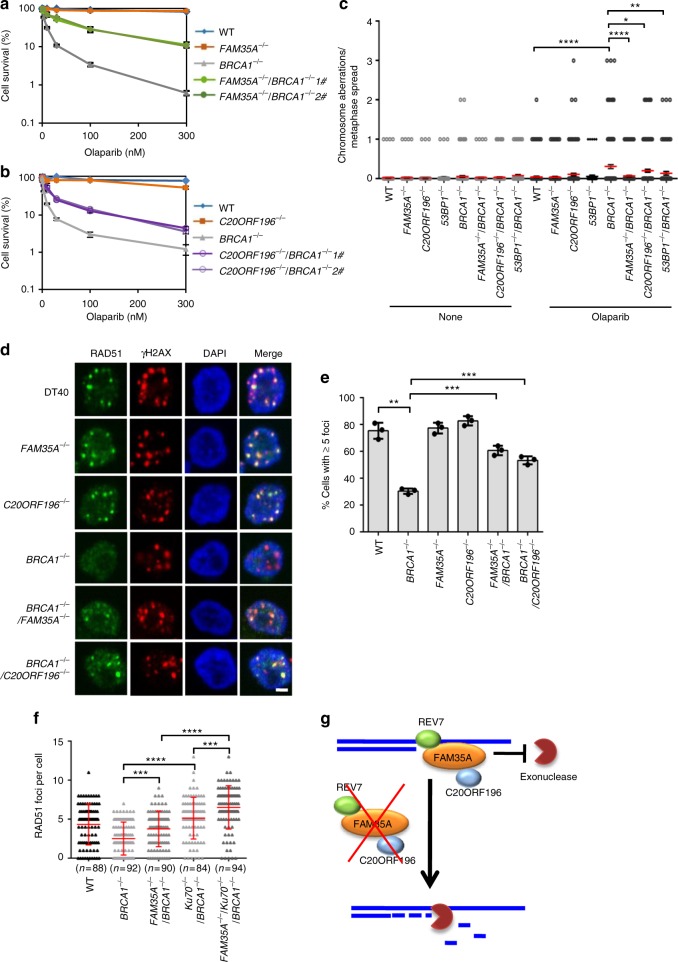
FAM35A and C20ORF196 block resection in the BRCA1-deficient cells. **a**, **b** The absence of FAM35A (**a**) or C20ORF196 (**b**) suppressed the olaparib sensitivity of the BRCA1-deficient cells. The mean and s.d. from three independent experiments are shown. **c** Chromosome aberration of various DT40 cells. Cells were treated with or without 1 μM olaparib for 8 h and then incubated with 100 ng ml^–1^ colcemid for 4 h before fixing. The mean and s.e.m. are shown. **d**, **e** Immunofluorescence (**d**) and its quantification (**e**) showing that FAM35A and C20ORF196 block the end resection in the BRCA1-deficient cells. The DT40 cells were treated with 4 Gy X-ray and released 4 h before fixing. The mean and s.d. from three independent experiments are shown. Scale, 2 μm. **f** A scatter diagram shows RAD51 foci numbers in DT40 cells after X-ray treatment. The mean and s.d. are shown. **g** A model shows that REV7–FAM35A–C20ORF196 complex blocks resection through binding the 5ʹ-recessed DSB ends. *p < 0.05; **p < 0.01; ***p < 0.001; ****p < 0.0001. Statistics was performed by two-tailed *t*-test

The 53BP1–RIF1–REV7 axis antagonizes BRCA1 to block DSB end resection and HR^10^. The absence of 53BP1, RIF1 or REV7 suppressed the PARP inhibitor (PARPi) sensitivity of the BRCA1-deficient cells^3–8,10^. PARPi disrupts the repair of ssDNA breaks, which are converted to one-end DSBs during replication and are toxic to the cells deficient of HR proteins, such as BRCA1. We examined whether the REV7–FAM35A–C20ORF196 complex has similar function and generated *FAM35A*^*−/−*^*BRCA1*^*−/−*^ and *C20ORF196*^*−/−*^*BRCA1*^*−/−*^ double knockout DT40 cells. *BRCA1*^*−/−*^ DT40 cells are hypersensitive to PARPi as reported^4^. Interestingly, the disruption of *FAM35A* or *C20ORF196* gene suppressed the PARPi sensitivity of the *BRCA1*^*−/−*^ cells (Fig. 5a, b). Moreover, analysis of chromosome aberration was consistent with these results (Fig. 5c), suggesting that FAM35A and C20ORF196 have BRCA1 antagonistic function in DSB repair similar to that of 53BP1, RIF1 and REV7. These results were further confirmed in human HCT116 cells (Supplementary Fig. 7a).

We further tested whether DNA end resection is altered in the absence of FAM35A or C20ORF196 in BRCA1-deficient cells. RPA binds to resected ssDNA and subsequently is phosphorylated by ATM^21^. Thus, RPA and phosphorylated RPA are widely used as a proxy for measuring resection. The signals of RPA and phosphorylated RPA were strongly decreased in the BRCA1-deficient HCT116 cells treated with IR, but recovered when FAM35A or C20ORF196 was depleted (Supplementary Fig. 7b–e), suggesting that FAM35A and C20ORF196 suppresses end resection in the *BRCA1*^*−/−*^ cells. RAD51 will accumulate on 3ʹ ssDNA overhang after end resection during HR^22^. After X-ray treatment, *BRCA1*^*−/−*^ DT40 cells showed significant decrease of RAD51 foci compared with wild-type cells (Fig. 5d, e). The disruption of *FAM35A* or *C20ORF196* gene largely suppressed this defect in the *BRCA1*^*−/−*^ DT40 cells (Fig. 5d, e), suggesting that FAM35A and C20ORF196 suppress end resection and HR in the BRCA1-deficient cells. Thus, FAM35A and C20ORF196 resemble 53BP1, RIF1 and REV7 to antagonize BRCA1 in DSB repair, indicating that REV7 performs its resection inhibition function through the REV7–FAM35A–C20ORF196 complex.

### FAM35A has non-redundant function with KU to block resection

KU70/80 complex has strong binding affinity to blunt DSB ends and its binding protects ends from resection^23–25^. However, because the low binding affinity of KU70/80 to ssDNA, it could not protect the 5ʹ-recessed ends efficiently after partial resection^1^. We speculated that FAM35A may provide an opportunity to protect partially resected DSB ends when KU70/80 sequesters. Disruption of KU70 gene strongly rescues RAD51 foci in the BRCA1-deficient cells after X-ray treatment, suggesting that KU70/80 protects the DSB ends from resection (Fig. 5f). Interestingly, disrupting both KU70 and FAM35A genes led to a higher level of RAD51 foci than single gene knockout in the BRCA1-deficient cells (Fig. 5f). These results suggest that KU70/80 and FAM35A have non-redundant functions to prevent DNA end resection, consistent with our speculation.

## Discussion

Altogether, our data identify a complex constituted of REV7–FAM35A–C20ORF196 as an important regulator of DSB repair pathway choice, which is downstream of 53BP1–RIF1 to antagonize BRCA1-dependent HR and to promote NHEJ. The complex is recruited to DSB sites mainly by RIF1 and binds preferably to ssDNA through the OB-folds of FAM35A (Fig. 2). FAM35A is structurally similar to POT1, which binds telomeric 3ʹ overhangs and blocks hyper-resection by Apollo/SNM1B exonuclease^15^. We speculate that FAM35A works at DSB ends in a manner to POT1 at telomeres (Fig. 5g). FAM35A is able to inhibit unscheduled resection initiated by CtIP-MRN, limit over-resection by EXO1/DNA2, or protect the recessed DSB ends, which might be a native product when DNA is damaged and is unable to be efficiently protected by KU70/80. A resection-dependent canonical NHEJ pathway was reported recently^26^. More recently, when our article was being prepared, several groups reported that FAM35A complex recruits CST(CTC1-STN1-TEN1)–Polα complex to DSB sites to protect the broken ends^27–33^ through mimicking the action of POT1 at telomeres, consistent with our hypotheses.

## Methods

### Cell culture and transfection

U2OS and HEK293 cells were cultured in Dulbecco’s modified Eagle’s medium (DMEM) medium containing 10% fetal bovine serum (FBS; Invitrogen). HEK293 suspension cells were cultured in SMM 293-TI medium (Sino Biological Inc.) supplemented with 1% Gibco FBS and 1% glutamine in an incubator with shaking at 140 r.p.m. The cell lines studied were obtained from the ATCC and are not among those listed as commonly misidentified by the International Cell Line Authentication Committee. All cell lines were subjected to mycoplasma testing twice per month and found to be negative. The identity of the cell lines was validated by Short Tandem Repeat profiling (ATCC) and by analysis of chromosome number in metaphase spreads.

HEK293 suspension cells were transfected with PEI. U2OS, HEK293T and HCT116 cells were transfected with FugeneHD (Promega). The small interfering RNAs (siRNAs) targeting Rif1 (RIF1siRNA in Supplementary Table 1), FAM35A (FAM35AsiRNA in Supplementary Table 1) and C20orf196 (C20ORF196siRNA in Supplementary Table 1), were transfected using RNAi MAX (Invitrogen). To produce the REV7 short hairpin RNA (Supplementary Table 1) virus, lentiviral plasmids were co-transfected into HEK293T cells using polyethylenimine (PEI). After 4 days, the supernatants containing the packaged lentivirus were harvested and stored at −80 °C until further use.

DT40 cells were gift from Dr. Shunichi Takeda and were cultured in RPMI-1640 medium supplemented with 10% fetal calf serum, 1% chicken serum, 10 mM HEPES and 1% penicillin–streptomycin mixture at 39.5 ℃, 5% CO_2_. Transfection was performed by electroporation using the LonzaNucleofector 4D. For selection, growth medium containing G418 (2 mg ml^−1^), puromycin (0.5 μg ml^−1^), Blasticidine (25 μg ml^−1^) or histidinol (1 mg ml^−1^) was used.

### Antibodies

Anti-FAM35A rabbit polyclonal antibodies (1:500 for Western-blotting [WB]) are purchased from Abcam (ab136079), anti-C20ORF196 rabbit polyclonal antibodies (1:500 for WB) from Sigma-Aldrich (hpa040749), anti-REV7 rabbit polyclonal antibodies (1:2000 for WB) from BD Biosciences (612266), anti-RAD51 rabbit polyclonal antibodies (1:200 for immunofluorescence [IF]) from Abcam (ab133534), anti-POLD3 rabbit polyclonal antibodies (1:2000 for WB) from Abnova (H00010714-M01), anti-GFP rabbit polyclonal antibodies (1:1000 for WB) from Proteintech Group (66002-1-Ig), anti-β-actin mouse monoclonal antibody (1:5000 for WB) from MBL (M177-3), anti-FLAG M2 monoclonal antibody (1:5000 for WB) from Sigma-Aldrich (F3165), anti-RPA32 rabbit monoclonal antibody (1:500 for IF) from Abcam (ab76420) and anti-pRPA32 (S4/S8) rabbit polyclonal antibodies (1:500 for IF) from Bethyl (A300-245A).

### Immunoprecipitation and MBP-pulldown

The complementary DNAs of REV7, FAM35A and C20ORF196 genes from hORFemone (V8.1) were transferred to mammalian expression destination plasmid pDEST26-FLAG with LR reaction (Invitrogen).

For immunoprecipitation (IP), expression plasmids were transiently transfected to HEK293 suspension cells with polyethyleneimine. Cells were harvested 64 h after transfection and pellets were directly lysed with NTEN buffer (20 mM Tris-HCl [pH 7.5], 150 mM NaCl, 10% glycerol, 0.5% NP40, 10 mM NaF, 1 mM phenylmethylsulfonyl fluoride (PMSF), 1 μg ml^−1^ leupeptin, 1 μg ml^−1^ aprotinin). The lysates were ultra-centrifuged at 440,000 × *g* for 15 min and then the supernatant was incubated with anti-Flag M2-conjugated agarose beads for 3–4 h at 4 °C. The beads were washed four times with IP buffer (20 mM Tris-HCl [pH 7.5], 150 mM NaCl, MgCl_2_ 5 mM, 10% glycerol, 0.1% NP40, 1 mM DTT and 1 mM PMSF) and then incubated with IP buffer containing 400 µg ml^−1^ 3XFlag peptide for 1–2 h. Subsequently, the eluted complexes were analyzed by sodium dodecyl sulfate–polyacrylamide gel electrophoresis (SDS-PAGE) and mass spectrometry.

For MBP-pulldown, 30 μg of pDEST26–MBP–FAM35A_RBD or pDEST26–MBP–MRE11 was transfected into 30 ml of HEK293 suspension cells using polyethyleneimine. The cells were harvested after 3 days and lysed in 3 ml of NTEN buffer. After ultra-centrifugation at 440,000 × *g* for 15 min at 4 °C, the supernatant was incubated with amylose resins at 4 °C for 4 h. The beads were washed four times with NTEN buffer, and eluted with 50 μl of phosphate-buffered saline (PBS) containing 20 mg ml^−1^ Maltose.

### Immunostaining and immunoblotting

DT40 cells were cultured to 0.8 × 10^6^ cells ml^−1^ before the experiments. After washing with PBS, the cells were re-suspended with PBS containing 1% bovine serum albumin (BSA) and mixed with ninefold volume of PBS containing 3% para-formaldehyde, 0.3% Triton, 0.5% BSA and 2% sucrose. The cells were immediately spun to slides with Cyto-spinner at 272 × *g* for 8 min. After washing three times with PBS with 0.05% Tween-20, the cells were blocked with 5% BSA (Sigma) in PBS for 15 min. The primary antibodies were diluted in PBS containing 1% BSA and incubated with the cells for 90 min. After washing, secondary antibodies diluted in PBS containing 1% BSA were added to the cells for 30 min. The cells were washed three times and mounted with ProLong Gold antifade reagent with DAPI (Invitrogen). Images were acquired with an LSM710 confocal (Zeiss) using a 100 × /1.4 NA objector.

For immunoblotting, primary antibodies were incubated for 1.5 h at room temperature in PBS with 0.05% Tween-20 and 5% powder milk. Secondary peroxidase-coupled antibodies (1:5000; Jackson ImmunoResearch) were incubated at room temperature for 45 min. Enhanced chemiluminescence  was detected by using film. Uncropped immunoblots are available in Supplementary Fig. 8.

### Laser microirradiation

U2OS or HEK293 cells expressing GFP–FAM35A or GFP–C20ORF196 were cultured at 37 °C in CO_2_-independent medium (Invitrogen) containing 10% FBS in a temperature-controlled container in glass-bottom dishes (MatTek). Laser microirradiation was carried out with the Micro-Point Laser Illumination and Ablation System (ANDOR) coupled to a Leica DMI8 microscope with a 63 × CS2 oil immersion objective. Images were acquired with ANDOR IQ3 software through an ANDOR IXON camera with ANDOR Dragonfly system.

### Generation of RIF1 knockout HEK293 cells

RIF1-deficient HEK293 cells were generated using CRISPR. Briefly, guide sequences (RIF1sgRNA in Supplementary Table 1) were inserted into the pX330 vector^34^. The guide sequence-containing pX330 plasmids were transfected into HEK293 cells and single colonies were picked after 8–10 days of incubation. The genomic fragments of the *RIF1* gene were amplified by PCR using primers, RIF1g1 and RIF1g2 (Supplementary Table 1). The products were digested with T7 endonuclease. Colonies containing the expected PCR fragments were then sequenced and examined by western blotting.

### Protein purification

FAM35A_C (381–904 aa) was fused with MBP-tag and His_6_-tag at C-terminus and N-terminus, respectively. Protein was expressed in *E. coli* (*Transetta* cells, TransGen) using pCPD9 vector. Cells were grown at 37 °C until OD600 = 0.8, and were induced with 0.05% Isopropyl β-D-1-thiogalactopyranoside at 16 °C for 12 h. The cell pellet from 12-liter culture was lysed by French Press in 200 ml lysis buffer (40 mM Tris Cl, pH 8.0, 500 mM NaCl, 25 mM imidazole, 7% glycerol, 0.2 mM DTT and 1 mM PMSF, 1 μg ml^−1^ leupeptin and 1 μg ml^−1^ aprotinin). The lysate was centrifuged at 35,000 × *g* for 30 min and the supernatant was incubated with 3 ml Ni-beads (GE Healthcare) at 4 °C for 1 h. The beads were washed four times with lysis buffer. The proteins were eluted with 15 ml His-Elution Buffer (20 mM Tris Cl, pH 8.0, 160 mM NaCl, 400 mM imidazole, 7% glycerol, 1 mM DTT, 1 mM PMSF, 1 μg ml^−1^ leupeptin and 1 μg ml^−1^ aprotinin). The eluted proteins were incubated with 0.5 ml Amylose resins (NEB) at 4 °C for 1 h. After washing four times with washing buffer (20 mM Tris-HCl, 500 mM NaCl, 0.1% Triton X-100, 1 mM DTT), protein was eluted with MBP-Elution Buffer (20 mM Tris Cl, pH 8.0, 160 mM NaCl, 7% glycerol, 1 mM DTT and 30 mM maltose). The protein was then concentrated, flash-frozen and stored at –80 °C.

### Gel-shift assay

The DNA substrates were made by annealing oligos H60f and H60r, H60f1/2 and H60r (Supplementary Table 1), respectively. The 5ʹ ends of the oligo H60f and H60f1/2 were labeled with ^32^P using T4 polynucleotide kinase before annealing. In all, 5 nM ^32^P -labeled DNA subtracts and the indicated amount of proteins were incubated at 25 °C in 10 μl reaction buffer (20 mM HEPES at pH 7.5, 5 mM MgCl_2_, 100 mM KCl, 1 mM DTT, 0.05% Triton X-100, 100 μg ml^−1^ BSA and 5% glycerol) for 15 min. Reaction mixture was loaded and resolved on a 5% Tris-borate-EDTA (TBE) gel.

### Generation of the DT40 knockout strains

DT40 knockout constructs for FAM35A and C20ORF196 were generated as previously^35^ using MultiSite Gateway Three-Flagment Vector Construction Kit. The 5ʹ and 3ʹ arms were amplified from genomic DNA using the primers FAM35A_5ARM1/FAM35A_5ARM2 and FAM35A3_ARM1/FAM35A3_ARM2 (for FAM35A; Supplementary Table 1), and C20ORF196_5ARM1/C20ORF196_5ARM2 and C20ORF196_3ARM1/C20ORF196_3ARM2 (for C20ORF196; Supplementary Table 1). The knockout constructs were linearized before transfection. The primers FAM35A_g1/FAM35A_g2 (for FAM35A; Supplementary Table 1) and C20ORF196_g1/C20ORF196_g2 (for C20ORF196; Supplementary Table 1) were used for genomic DNA PCR. The generation of *RIF1*^*−/−*^ and *BRCA1*^*−/−*^ DT40 cells were described as previously^5,36^. *FAM35A*^*−/−*^*BRCA1*^*−/−*^ DT40 cells were generated by knockouting *BRCA1* gene in *FAM35A*^*−/−*^ cells. *FAM35A*^*−/−*^*RIF1*^*−/−*^ DT40 cells were generated by knockouting *FAM35A* gene in *RIF1*^*−/−*^ cells. *FAM35A*^*−/−*^ *Ku70*^*−/−*^*BRCA1*^*−/−*^ DT40 cells were generated by knockouting *KU70* and *BRCA1* genes successively.

### Cell survival assay

In total, 1500–3000 cells were plated into each well of 96-well plates with a range of doses of ICRF193, cisplatin or camptothecin (CPT). After 48 h of incubation, cells were pulsed with CellTiter 96 Aqueous One Solution Reagent (Promega) for 4 h. Cell viability was measured by luminometer, and each dose point was measured in triplicate. For Bleomycin and VP16, a density of 300–1000 cells per well and a 72-h incubation were used.

### Statistics

Statistics was performed by two-tailed *t*-test. The data were normally distributed and the variance between groups being statistically compared was similar. No statistical methods or criteria were used to estimate sample size or to include/exclude samples. The investigators were not blinded to the group allocation during the experiments.

## Electronic supplementary material

Supplementary Information

Peer Review File

Description of Additional Supplementary Files

Dataset 1

## Data Availability

All relevant data are available from the authors.
