# Supplementary material for: An OB-fold complex controls the repair pathways for DNA double-strand breaks
Source: Nat Commun. 2018 Sep 25;9:3925. doi: 10.1038/s41467-018-06407-7 (PMC6156606; doi:10.1038/s41467-018-06407-7)
Supplement: Supplementary file 1 — Supplementary Information [file 41467_2018_6407_MOESM1_ESM.pdf]

Title: A OB-fold complex controls the repair pathways for DNA double-strand breaks

Author: Gao, Feng et al.

SUPPLEMENTARY INFORMATION

The supplementary information contains 8 supplementary figures (including legends) and one supplementary table

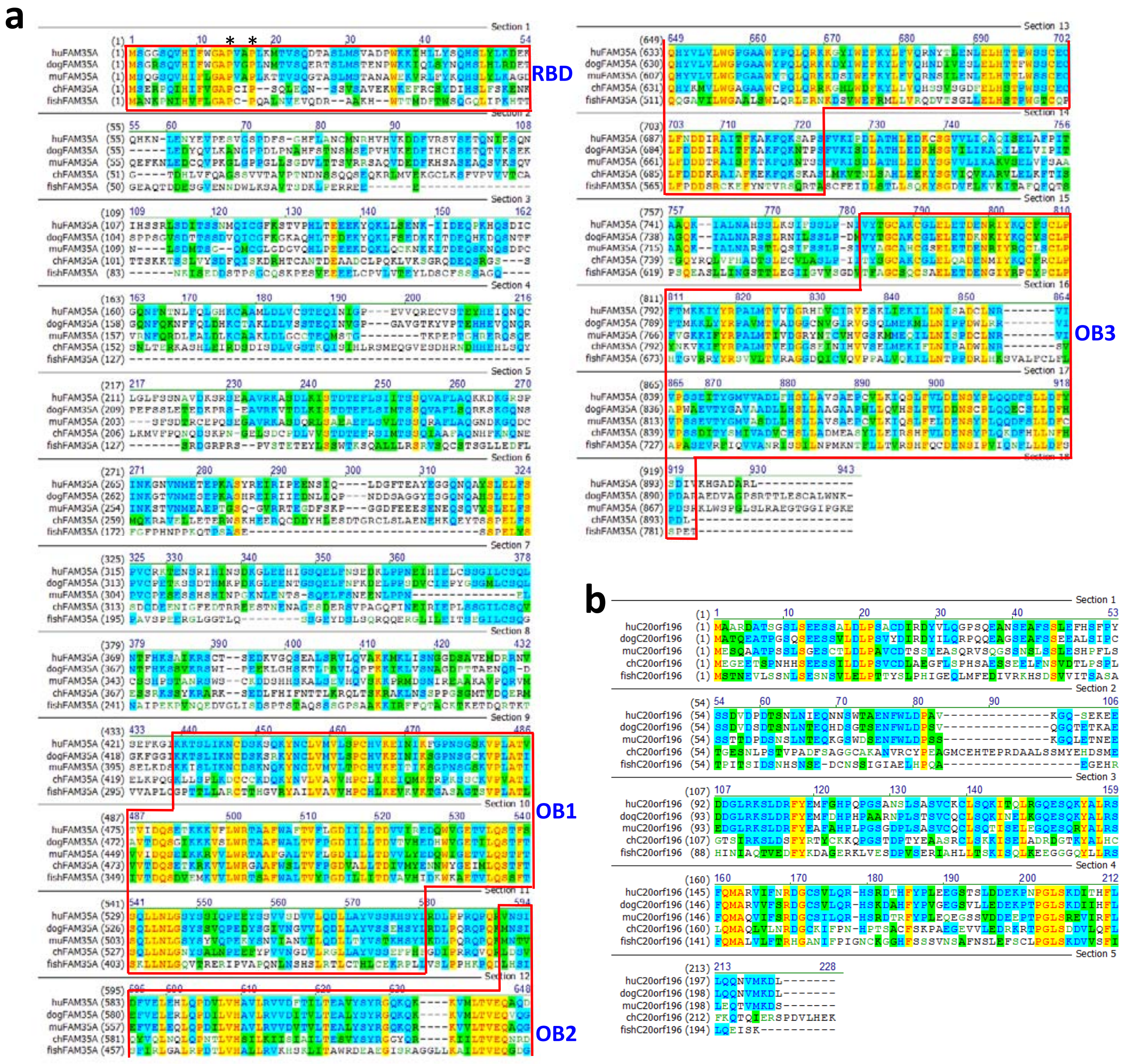

Supplementary Figure 1. Alignment. a, Alignment of FAM35A. FAM35A orthologues were identified using the BLASTP algorithm, which searches the NR database maintained by NCBI. Human (hu, *Homo sapiens* Q5RCM2.1), Dog (dog, *Canis lupus familiaris* XP_022273327.1), mouse (mu, *Mus musculus* NP_083665.2), chicken (ch, *Gallus gallus* XP_015143518.1) and zebrafish (fish, *Danio rerio* NP_001139105.1) orthologues are shown. The identical and conserved amino acids are highlighted with red letters on a yellow background and dark blue letters on a blue background, respectively, weakly similar amino acids are highlighted on a green background, and green letters indicate blocks of similar amino acids. Asterisks indicate mutated amino acids in P14A/P17A. **b**, Alignment of C20ORF196. Human (hu, *Homo sapiens* NP_001290406.1), Dog (dog, *Canis lupus familiaris* XP_022264881.1), mouse (mu, *Mus musculus* NP_001345190.1), chicken (ch, *Gallus gallus* XP_015139412.1) and zebrafish (fish, *Danio rerio* XP_020391945.1) orthologues are shown.

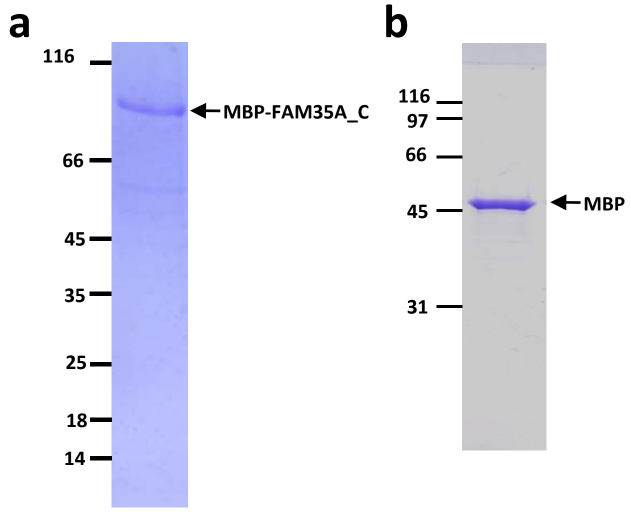

Supplementary Figure 2. Purified protein of MBP-FAM35A_C and MBP. **a**, **b**, 1μg MBP-FAM35A_C (a) and MBP (b) were loaded on SDS-PAGE and stained by Coomassie blue.

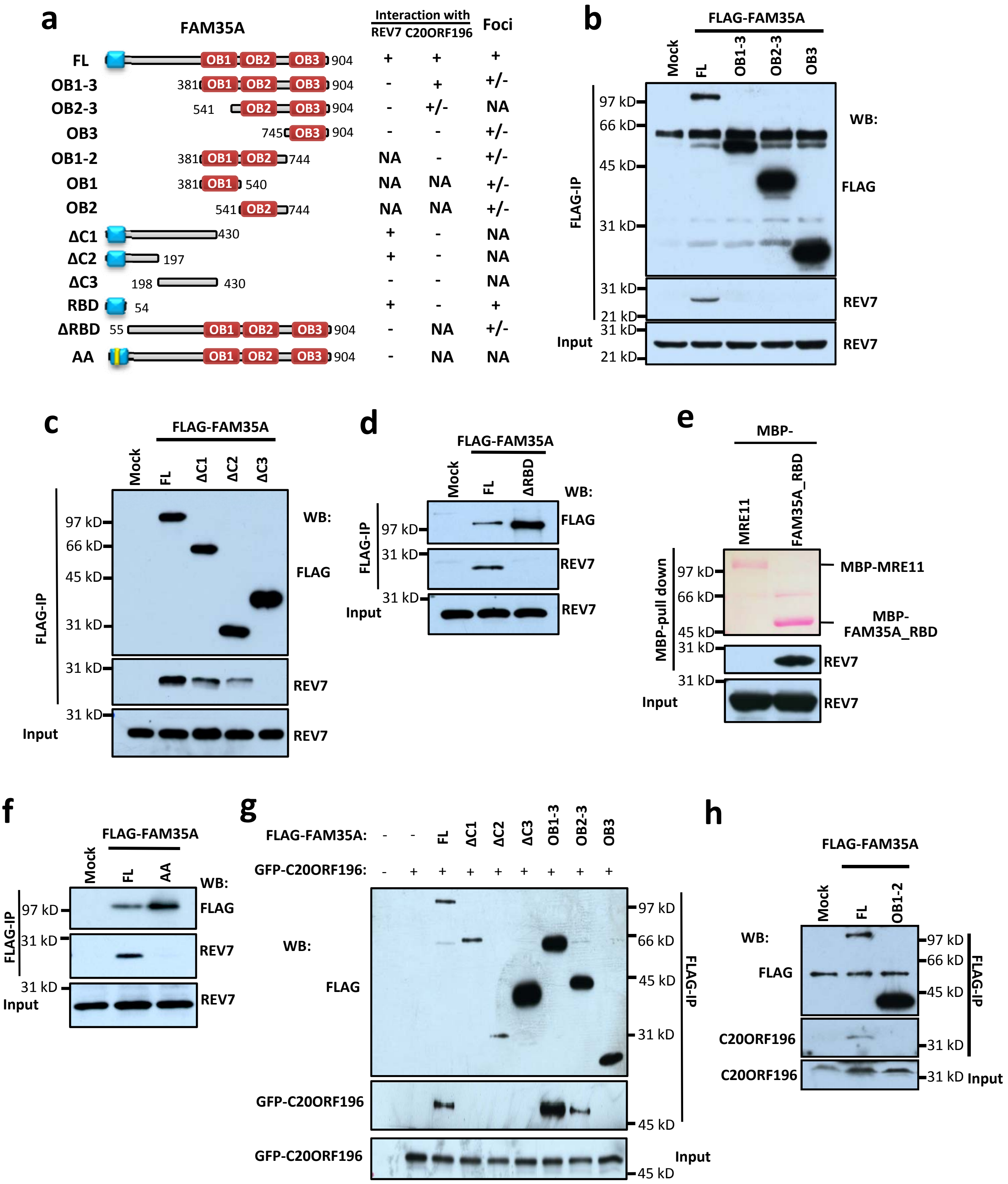

Supplementary Figure 3. FAM35A interacts with REV7 and C20ORF196 through its N-terminal and C-terminal region, respectively. a, Schematic representation of the different FAM35A deletion mutants (left) and their ability to co-immunoprecipitate with REV7 and C20ORF196 from HEK293 extracts and to re-localize to DSB sites (right). AA, P14A/P17A. **b-h**, Immunoprecipitation (b-d, f-h) and MBP-pull down (e) to assess whether the various deletion mutants of FAM35A described in (a) co-purified with REV7 (b-f) and C20ORF196 (g, h). MBP-fused proteins were stained by Ponceau S red. Other proteins were detected by Western-blotting. MBP-MRE11 was included as a control.

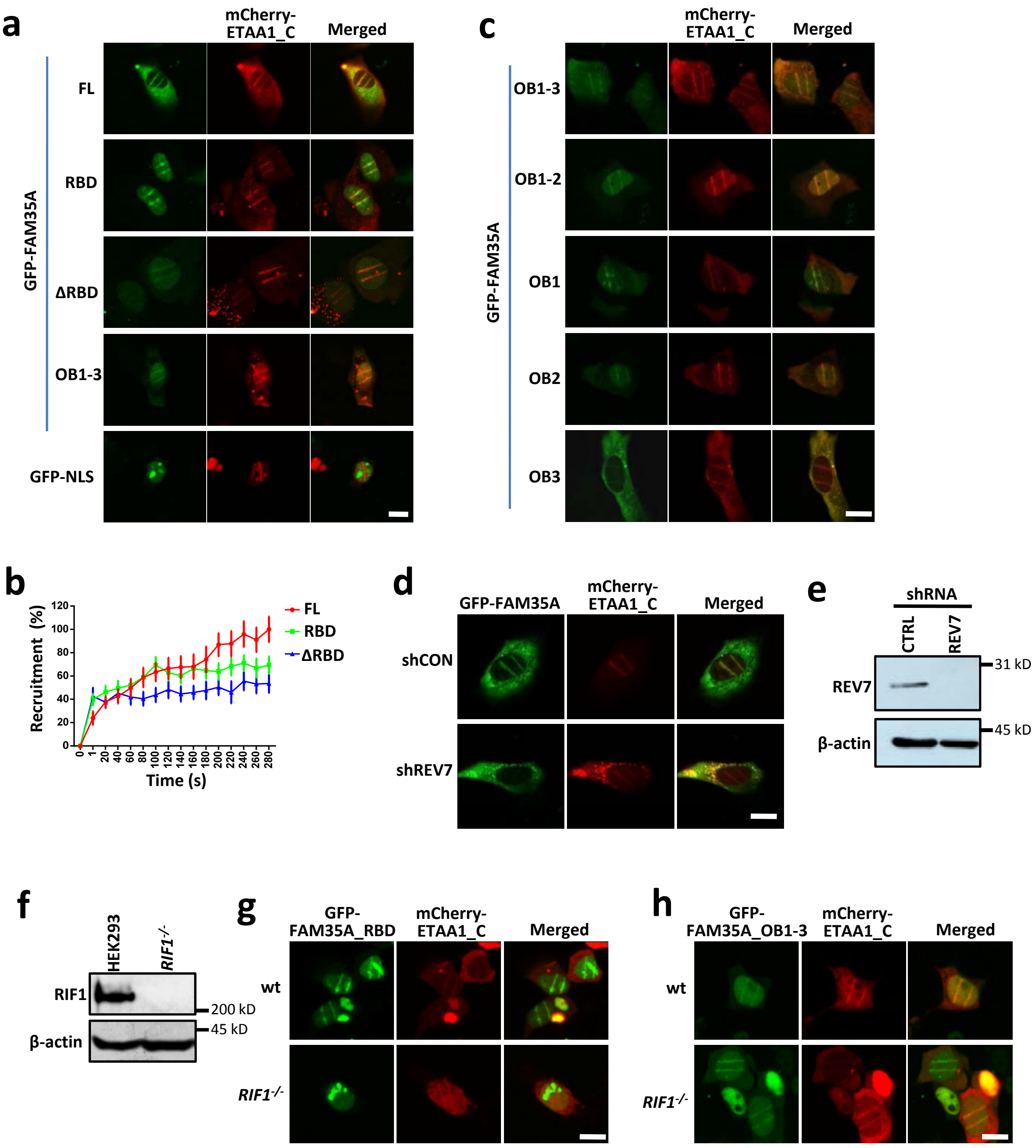

Supplementary Figure 4. REV7 recruits FAM35A to DNA damage sites. a-c, Recruitment of wild-type or truncated GFP-FAM35A to laser-induced DNA damage sites (a, c) and their quantifications (b). mCherry-fused ETAA1 C-terminal region (511-927aa) was used as a positive control. GFP fused with a nuclear localization signal peptide (GFP-NLS) was included as a negative control. Scale bar, 5 µm. For every sample, 25 cells were quantified. The mean and s.e.m of every time point are shown. **d**, Laser-induced foci of the GFP-tagged FAM35A in the REV7-depleted U2OS cells. **e**, Immunoblotting shows the efficiency of REV7 knockdown. **f**, Validation of the *RIF1*$^{-/-}$ HEK 293 cells by immunoblotting. **g**, **h**, Recruitment of FAM35A RBD domain (g) or OB1-3 region (h) in RIF1 deficient cells.

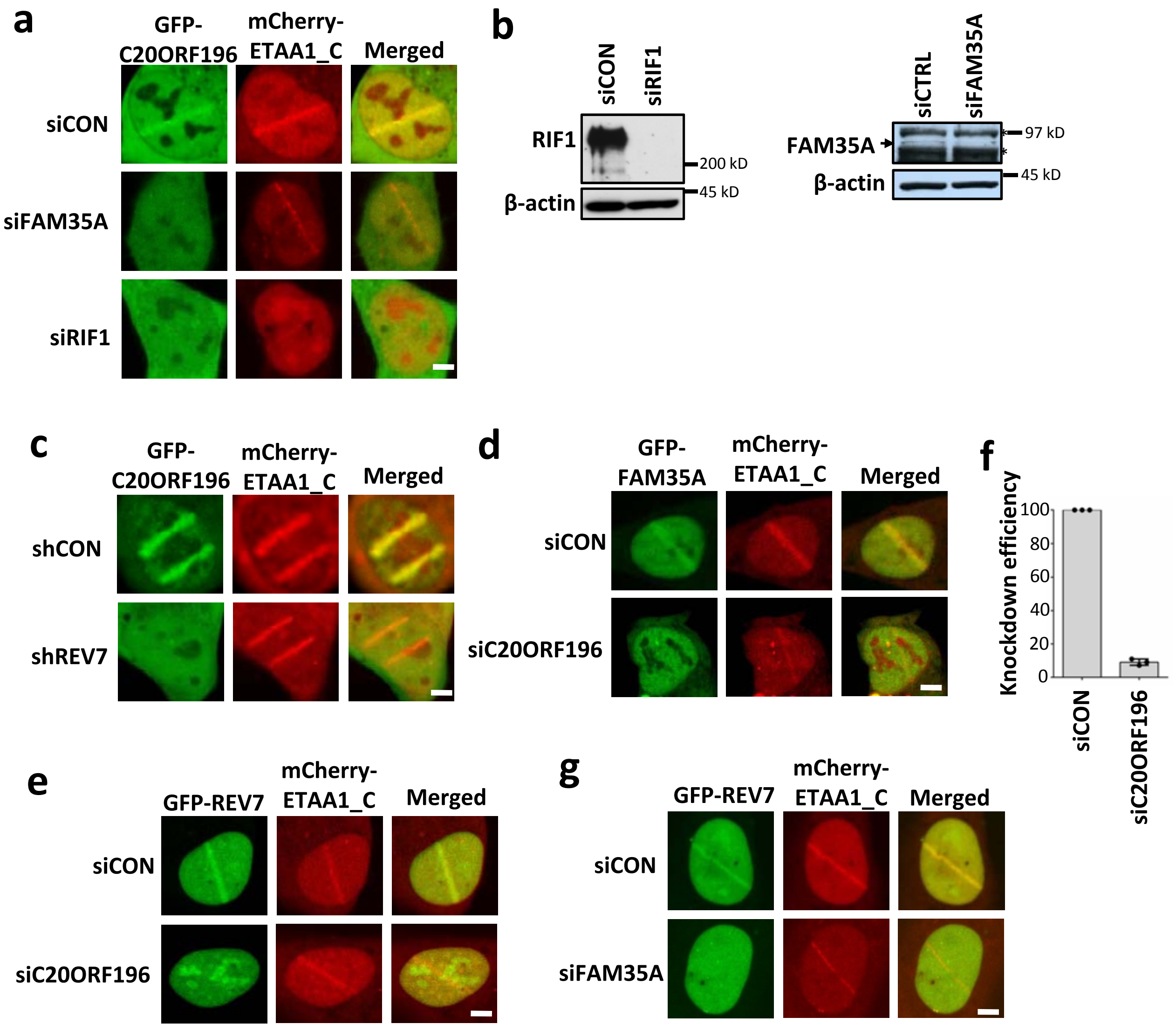

Supplementary Figure 5. C20ORF196 was recruited to DNA damage sites. **a**, **c**, Recruitment of C20ORF196 to laser-induced DNA damage sites when FAM35A, RIF1 (a) or REV7 (c) was depleted. Scale bar, 2 µm. **b**, Immunoblotting shows the knockdown efficiency of RIF1 and FAM35A. **d**, **e**, Laser-induced foci of the GFP-tagged FAM35A (d) and REV7 (e) in the C20ORF196 depleted U2OS cells. **f**, qPCR showing the mRNA level of C20ORF196 after knockdown. The mean and s.d. from three independent experiments are shown. **g**, Laser-induced foci of the GFP-REV7 in the FAM35A-depleted cells.

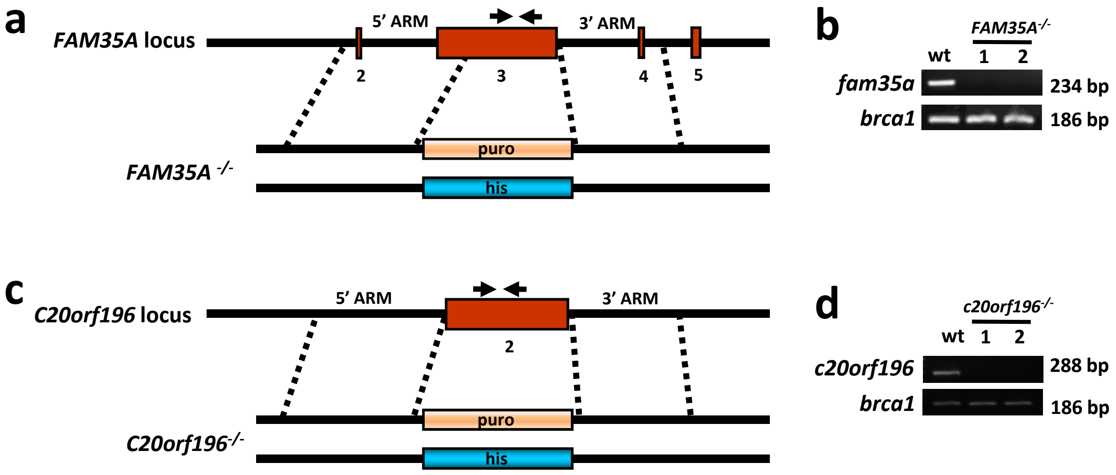

Supplementary Figure 6. Generation of FAM35A and C20orf196 knockout DT40 cells. a, **c**, Schematic representation of the chicken wild-type and targeted genomic DNA in the *FAM35A* (a) and *C20ORF196* (c) gene. The regions containing exons (marked by red) of these genes are replaced by histidinol or puromycin resistant gene. The two regions between two pairs of dotted lines were used as arms of the knock-out constructs. Arrows indicated targeted locations of primers for genomic PCR. **b**, **d**, Genome-PCR analysis to show that *FAM35A* (b) and *C20orf196* (d) genes are undetectable in the respective knockout DT40 cells. *brca1* was included as a positive control.

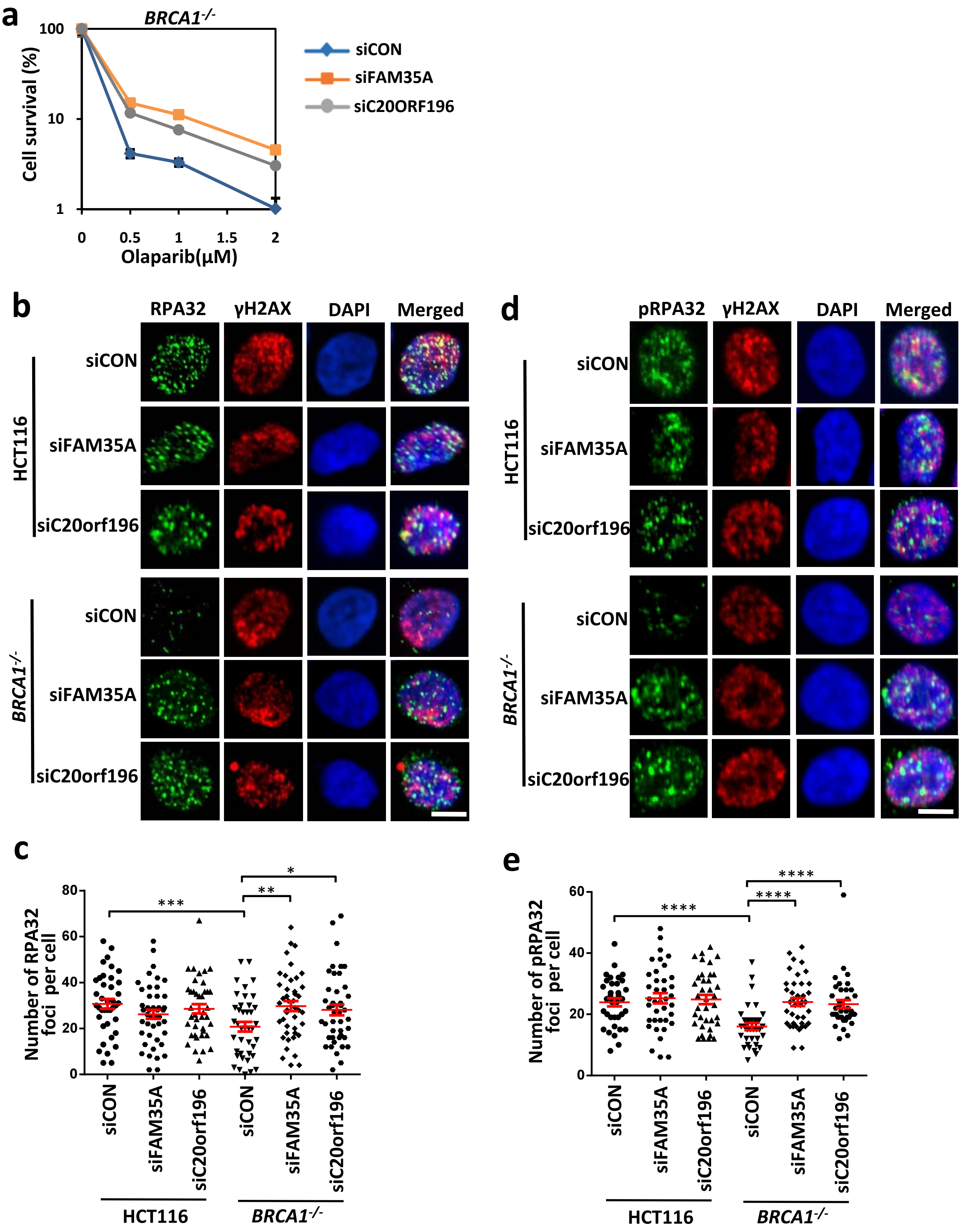

Supplementary Figure 7. The absence FAM35A or C20orf196 suppresses the olaparib-sensitivity of BRCA1-deficient human cells. a, Olaparib sensitivity assay of *BRCA1*$^{-/-}$ HCT116 cells after FAM35A or C20orf196 knockdown. **b**-**e**, Immunofluorescence (b, d) and their quantification (c, e) of RPA32 (b, c) and pRPA32 (RPA32-S4/8p) (d, e) foci. Cells were treated with 25 Gy X-ray 1 hr before staining. Scale bar, 5 µm. *, p<0.05; **, p<0.01; ***, p<0.001; ****, p<0.0001. Statistics was performed by two-tailed *t*-test

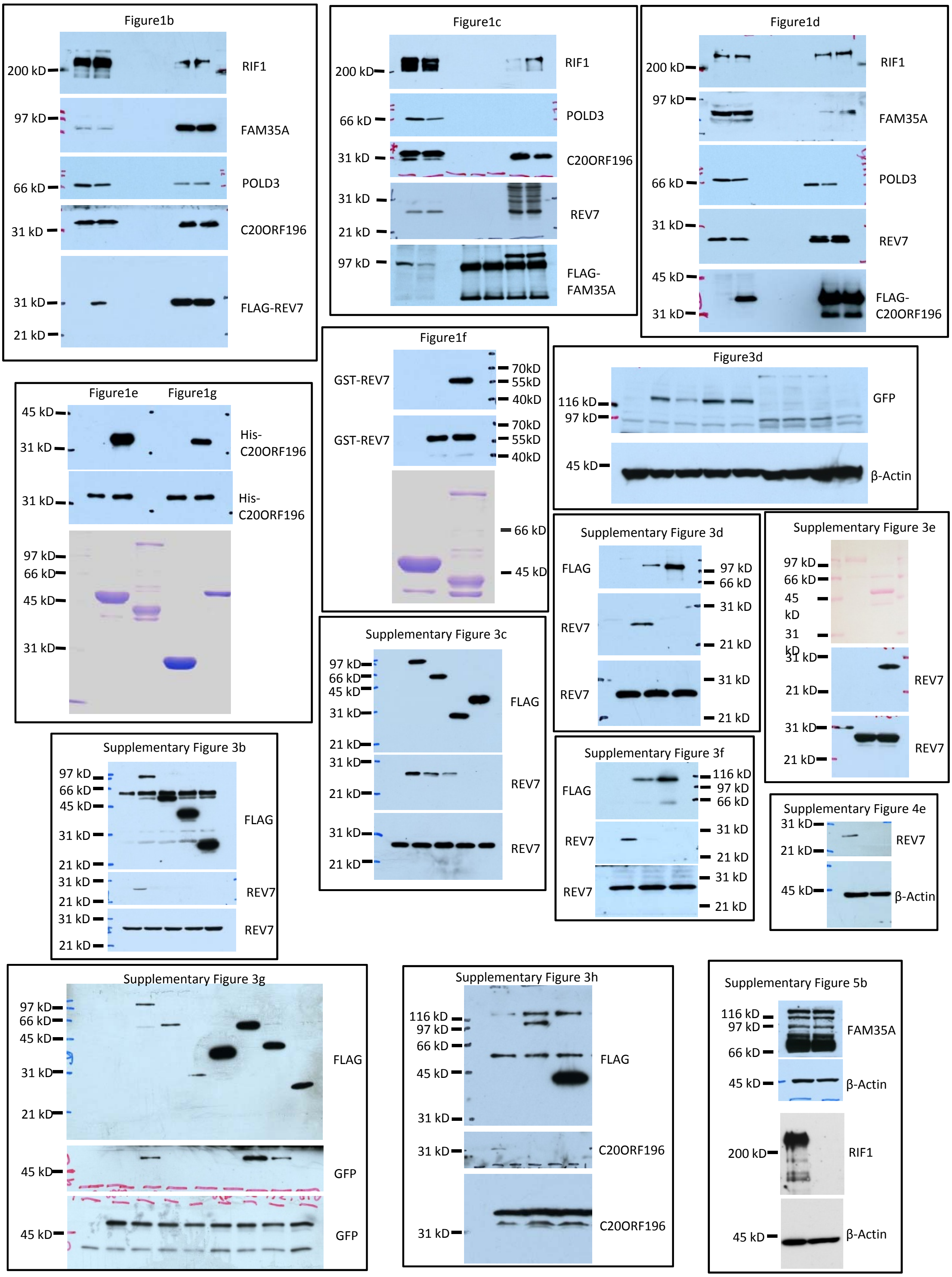

Supplementary Figure 8. Uncropped immunoblots. Unprocessed images of scanned immunoblots shown in Figure 1b-g, Figure 3d, Supplementary Figure 3b-h, Supplementary Figure 4e and Supplementary Figure 5b of the manuscript are provided.

Supplementary Table 1. Oligos.

Oligo name	Sequence	
RIF1siRNA	GCAGCUUAUGACUACUAAA	RIF1 siRNA
FAM35AsiRNA	AAGGAGUGGTTCUGAUUAA	FAM35A siRNA
C20ORF196siRNA	GCAGCAGAAUGUAAUGAAA	C20ORF196 siRNA
REV7shRNA	CCGGCACTCGCAACATGGAGAAGATC TCGAGATCTTCTCCATGTTGCGAGTGT TTTT	REV7 shRNA
RIF1sgRNA-f	CCCCATACCCTACAAGTCGG	RIF1 sgRNA forward
RIF1sgRNA-r	CCGACTTGTAGGGTATGGGG	RIF1 sgRNA reverse
RIF1g1	GTGGTTCTCTTGACAAAACCAGTC	RIF1 genome PCR forward primer
RIF1g2	GTCCCTTCTCTTTGCTTTGCTTTTG	RIF1 genome PCR reverse prime
H60f	GACGCTGCCGAATTCTACCAGTGCCTT GCTGGACATCTTTGCCCACCTGCAGGT TCACCC	Substrate for DNA binding assay
H60r	GGGTGAACCTGCAGGTGGGCAAAGAT GTCCAGCAAGGCACTGGTAGAATTCGG CAGCGTC	Substrate for DNA binding assay
H60f1/2	GACGCTGCCGAATTCTACCAGTGCCTT GCT	Substrate for DNA binding assay
FAM35A_5ARM1	CATCAGGCTCAGCACTGCCA	For FAM35A knockout in DT40 cells
FAM35A_5ARM2	GGAATACAGGGTGCTCCAACA	For FAM35A knockout in DT40 cells
FAM35A_3ARM1	GAGCCCGCAAGTCTGAAGAT	For FAM35A knockout in DT40 cells
FAM35A_3ARM2	CCCCTCTTGAAAACATCCCA	For FAM35A knockout in DT40 cells
C20ORF196_5ARM1	GCGGTAAGGTTATGTGCTTGTA	For C20ORF196 knockout in DT40 cells
C20ORF196_5ARM2	TCAAAGAGGTGGAAACTGGGA	For C20ORF196 knockout in DT40 cells
C20ORF196_3ARM1	TAGAATGGCTTAGGTTAGAGGGGT	For C20ORF196 knockout in DT40 cells
C20ORF196_3ARM2	CTCCAAACAGAAATGCTGAAGA	For C20ORF196 knockout in DT40 cells
FAM35A_g1	AGGTACTGACCATTTAGTGTTTCAG	For FAM35A knockout in DT40 cells
FAM35A_g2	GAAAACAAAGGTACTGACCATTTAG	For FAM35A knockout in DT40 cells
C20ORF196_g1	AGTAGCACCACCTGAAGAGCA	For C20ORF196 knockout in DT40 cells
C20ORF196_g2	CTTGTGTGTGTGTTTTTTCTGCTG CTTGTGTGTGTGTTTTTTCTGCTG	For C20ORF196 knockout in DT40 cells

Oligo sequences for siRNAs, shRNAs, sgRNAs and primers are listed.
