# Supplementary material for: An OB-fold complex controls the repair pathways for DNA double-strand breaks
Source: Nat Commun. 2018 Sep 25;9:3925. doi: 10.1038/s41467-018-06407-7 (PMC6156606; doi:10.1038/s41467-018-06407-7)
Supplement: Supplementary file 3 — Description of Additional Supplementary Files [file 41467_2018_6407_MOESM3_ESM.pdf]

Description of Additional Supplementary Files

File Name: Supplementary Data 1

Description: Mass spectrometry analysis of the mixtures of the indicated immunoprecipitates
