# Supplementary material for: An OB-fold complex controls the repair pathways for DNA double-strand breaks
Source: Nat Commun. 2018 Sep 25;9:3925. doi: 10.1038/s41467-018-06407-7 (PMC6156606; doi:10.1038/s41467-018-06407-7)
Supplement: Supplementary file 2 — Peer Review File [file 41467_2018_6407_MOESM2_ESM.pdf]

This manuscript has been previously reviewed at another journal that is not
operating a transparent peer review scheme. This document only contains reviewer comments and rebuttal letters for versions considered at Nature Communications .

REVIEWERS' COMMENTS:

Reviewer #1 (Remarks to the Author):

The authors have satisfactorily addressed my concerns.

REVIEWERS' COMMENTS:

Reviewer #1 (Remarks to the Author):

The authors have satisfactorily addressed my concerns.

Re: We thank this reviewer for his/her positive comment!
